# Proteomic Analysis of Protective Effects of Dl-3-n-Butylphthalide against mpp + -Induced Toxicity via downregulating P53 pathway in N2A Cells

**DOI:** 10.1186/s12953-022-00199-x

**Published:** 2023-01-03

**Authors:** Yuan Zhao, Jian Zhang, Yidan Zhang, Shuyue Li, Ya Gao, Cui Chang, Xiang Liu, Lei Xu, Guofeng Yang

**Affiliations:** 1 Department of Geriatrics, The Second Hospital of Hebei Medical University, Shijiazhuang, Hebei, People’s Republic of China; 2 Department of Neurology, The Second Hospital of Hebei Medical University, Shijiazhuang, Hebei, People’s Republic of China

**Keywords:** Parkinson’s disease (PD), Dl-3-n-butylphthalide (NBP), Neuroprotection, Tandem mass tags (TMTs), Proteomics, P53 signaling pathway

## Abstract

**Background:**

Dl-3-n-butylphthalide (NBP) is an important medial therapy for acute ischemic stroke in China. Recent studied have revealed that NBP not only rescued the loss of dopaminergic neurons in cellular and animal models of Parkinson's disease (PD), but also could improve motor symptoms in PD patients. However, the protective mechanism is not fully understood. P53 is a multifunctional protein implicated in numerous cellular processes, including apoptosis, DNA repair, mitochondrial functions, redox homeostasis, autophagy and protein aggregations. In PD, p53 integrated with various neurodegeneration-related signals inducing neuronal loss, indicating the suppression of P53 might be a promising target for PD treatment. Therefore, the purpose of the current study was to systemically screen new therapeutic targets of NBP in PD.

**Method:**

In our study, we constructed mpp + induced N2A cells to investigate the benefit effect of NBP in PD. MTT assay was performed to evaluate the cell viability; TMT-based LC–MS/MS was applied to determine the different expressed proteins (DEPs) of NBP pretreatment; online bioinformatics databases such as DAVID, STRING, and KEGG was used to construe the proteomic data. After further analyzed and visualized the protein–protein interactions (PPI) by Cytoscape, DEPs were verified by western blot.

**Result:**

A total of 5828 proteins were quantified in the comparative proteomics experiments and 417 proteins were considered as DEPs (fold change > 1.5 and *p* < 0.05). Among the 417 DEPs, 140 were upregulated and 277 were downregulated in mpp + -induced N2A cells with NBP pretreatment. KEGG pathway analysis indicated that lysosome, phagosome, apoptosis, endocytosis and ferroptosis are the mainly enriched pathways. By using MCL clustering in PPI analysis, 48 clusters were generated and the subsequent KEGG analysis of the top 3 clusters revealed that P53 signaling pathway was recognized as the dominant pathway for NBP treatment.

**Conclusion:**

NBP significantly relived mpp + -induced cell toxicity. The neuroprotective role of NBP was implicated with P53 signaling pathway in some extent. These findings will reinforce the understanding of the mechanism of NBP in PD and identify novel therapeutic targets.

**Supplementary Information:**

The online version contains supplementary material available at 10.1186/s12953-022-00199-x.

## Introduction

Parkinson’s disease (PD) is a multi-factorial age-related disorder which is characterized by irreversible impairment of normal movement coordination. Its cardinal motor impairment features are tremor, gait rigidity, bradykinesia and hypokinesia [1, 2]. The hallmark of PD pathology is the formation of Lewy bodies (LBs), which is consisted of misfolded and fibrillary forms of α-synuclein (α-syn) in surviving neurons [3]. At present, PD affect almost 1–2% of the world population, whereas the estimated prevalence rate would be double in 2040 [4–6]. Based on previous studies, the complicated mechanisms for PD development were ascribed to mitochondrial dysfunction, oxidative stress, apoptosis and neuroinflammation [3, 7, 8]. To date, many efforts have been made to explore the potential mechanism to counteract PD, however, it is still lack efficient therapy.

DL-3-n-butylphthalide (NBP), the first class I novel drug, which has been approved for the treatment of acute ischemia stroke in China since 2002 [9–11]. On account of the neuroprotective properties of NBP by eliminating free radicals, restoring mitochondrial function, reducing neuroinflammation and alleviating neuronal apoptosis, its therapeutic spectrum has expanded to various neurodegenerative diseases, such as Alzheimer disease (AD), Amyotrophic Laternal Sclerosis (ALS) and PD [12–16]. To date, many studies have clarified the benefit role of NBP in PD both in vitro and in vivo. Wang et al., demonstrated that NBP could rescue dopaminergic neurons by restoring mitochondrial function and alleviating NLRP3-mediated neuroinflammation [17]. It revealed NBP protected dopamine neurons by preventing the generation of ROS as well [18]. A preclinical study suggested the favorable effect of NBP in improving bradykinesia plus rigidity through UPDRS III motor evaluation [19]. However, there is still lack a systemic analysis to provide an insight into the protein profiling and functional pathways after NBP treatment in PD models.

Proteins are the crucial agent to execute various cell functions encoded by different genome, whereas the generation of high-quality protein expression profiling is much lagged behind RNA/DNA expression profiling [20, 21]. Traditionally, proteomic studies are engaged to investigate a large-scale of protein expressions, in order to provide new insight of the protein interactions, cellular functions and biological framework [22, 23]. Mass spectrometry (MS)-based quantitative proteomic studies using isobaric tags (eg., tandem mass tags, TMT; isobaric tags for absolute and relative quantification, ITRAQ) are well-established to identify differential expressed peptides with few missing values and precise quantification [2, 24, 25]. In addition, the quantified peptides are matched or identified using automated database searching (Uniprot is selected in our research) [26]. Biological function analysis is conducted through an online tool (Database for Annotation, Visualization, and Integrated Discovery, DAVID) and protein interactions are assayed through The Search Tool for the Retrieval of Interacting Genes/Proteins (STRING) [27]. Therefore, the reliable and deep-investigated protein data is well for discovering new therapeutic targets and the implicated biological pathways.

In the present study, we employed TMT-labeled global quantitative proteomic analysis to explore the DEPs in mpp + -induced N2A cells with or without NBP pretreatment. The biological functions and entirely canonical pathways of DEPs were conducted by GO, KEGG and protein–protein interaction (PPI) networks. As a result, we leverage the data to improve the understand of the neuroprotective role of NBP and its potential therapeutic mechanisms.

## Materials and methods

### Cell culture

The N2A cells which donated by Ji Jianguo lab from Peking University, were cultured in DMEM (Hyclone, USA)) containing 5% FBS (Hyclone, USA). The cells were settled in a 37 °C incubator with a humidified 95% air and 5% CO_2_. The cells were seeded in the 12-well microplates at a density of 1 × 10^4^ cells/ml. We changed the culture medium every 1–2 days. The experimental groups were arranged as follows, control group (without NBP and MPP + treatment); MPP + -treated group (500 μM MPP + treated for 24 h); (3) NBP + MPP + treated group (5 μM NBP treated for 27 h and 500 μM MPP + for 24 h).

### MTT assays to evaluate cell viability

The N2A cells were seeded in 96-well plates at a density of 1000 cells/well. Until the cells reached 50–60% confluence, different treatments were performed to the cells. Firstly, cells were maintained with 0, 40, 100, 250, 500 or 1000 μM MPP + for 24 h to detect the optimal concentration of MPP. Next, we selected different concentration of NBP (0, 0.5uM, 1uM, 2uM, 5uM and 10uM) for N2A cells treatment alone for 24 h to evaluate the cell toxicity of NBP. Last but not the least, cells were pretreated with varies concentration of NBP (0, 0.5uM, 1uM, 2uM, 5uM and 10uM) for 3 h and incubated with 500 μM MPP + for anther 24 h. MTT assay was carried out according to the manufacturer’s introduction (Beyotime Biotechnology, Shanghai, China). Briefly, MTT (5 mg/ml) solvent was added into each well (10 µl) and incubated at 37◦C for 4 h. After discarded the supernatant, 150 μL of DMSO was added into each well to dissolve the resulting MTT formazan. The absorbance at 570 nm was read by a microtiter plate reader (Multiskan FC, Thermo, USA).

### Protein extraction and preparation

The N2A cells with different treatments were harvested and recovered by centrifugation (1000xg for 5 min at 4 °C). Four volumes of pre-chilled acetone were added into protein extractions overnight at − 20◦C to obtain precipitation. The protein samples were stored at − 80◦C for further analysis.

### Trypsin/LysC Protein Digestion

60 μL 8 M urea was added to the precipitated proteins. The constructed protein suspensions were further broken using a Bioruptor Sonication Device. After determining the concentration of each protein sample using BCA assay, 60 ug protein sample was alkylated by incubation with 3 μL of 100 mM dithiothreitol (DTT; Sigma Aldrich) and 3 μL of 200 mM iodoacetamide (IAA; Sigma Aldrich) for 30 min at room temperature. Next, samples were digested into peptides by using lys-C (1:100 dilution) and trypsin (1:50 dilution) at 37◦C overnight.

### Peptides cleanup

The digested peptides were acidified with Trifluoroacetic Acid (TFA) to a final concentration of 0.5% (pH was around 2–3). After that, samples were desalted sequentially for 1 min in small 1 ml C18 Sep-Pak columns (3 M EmporeTM, CA, USA) with acetonitrile (ACN), 0.1%TFA/70%ACN and 0.1% TFA followed by conditioning with methanol for 1 min. Finally, the desalted peptide mixture was dried by vacuum centrifugation via speedvac.

### TMT-labeling

The peptides were resuspended with 100 mM tetraethylammonium bicarbonate (TEAB) and the concentration of peptides was determined using the Quantitative Colorimetric Peptide Assay (Cat. No. 23275, Thermo, USA). The TMT labeling reagent (0.8 mg) was dissolved in 41 μL of ACN. 12 μg peptides of each sample was labeled with 9 μL of the different TMT regent at room temperature for 60 min followed by quenching with eight microliter of 5% hydroxylamine for 20 min. In our study, we conducted three independent experiments, samples were labeled with TMT with reporter ions at m/z = 126, 128 (experiment 1); 127, 129 (experiment 2) and 127, 128 (experiment 3). In each experiment, the labeled channels were combined and dried down via speedvac.

### Peptide fractionation

The combined TMT-labeled samples were dissolved in 1% formic acid and desalted in C18 Sep-Pak columns. Gradient elution was performed with 0.1% ammonium hydroxide (pH 10) (reagent A) and ACN (reagent B). The gradient conditions for the fractionation were 90% A/10% B, 87.5% A/12.5% B, 85%A/15% B, 82.5% A/17.5% B, 80% A/20% B, 77.5% A/22.5% B, 75% A/25% B, and 50% A/50% B. Therefore, peptides were eluted with the above solvents into seven fractions and dried via speedvac.

### LC–MS/MS and data process

Samples were run on a Orbitrap Fusion Lumos mass spectrometer (Thermo, USA). a full MS survey scan (300–1500 m/z) was acquired at a resolution of 120,000 (at 200 m/z). The settings of the automatic gain control (AGC) target for MS1, maximum injection time, and a radio frequency (RF) lens were set as 1 × 106, 100 ms, and 30% respectively. The abundant ions with a charge state ≥ 2 were isolated in a 3 s cycle time. High-energy collision dissociation (HCD) MS/MS scans were set as follows: 37% collision energy, a mass resolution of 50,000, normalized AGC target at 1 × 105, isolation width of 1.2 m/z, dynamic exclusion at 30 s, and 10 parts per million (ppm) mass window.

The raw data was analyzed by using the SEQUEST algorithm implemented in the search engines: Proteome Discoverer (Version 2.2, Thermo Fisher Scientific) and searched in UniProt Database against the mus musculus FASTA files (August, 2013). The Sequest search parameters were include: trypsin digestion with two missed cleavages allowed; fixed modification, carbamidomethyl of cysteine; variable modification, oxidation of methionine, 10 ppm and 0.5 Da for MS tolerance; and a false discovery rate (FDR) < 1%. Protein identification required at least one unique peptide per protein group. Protein quantification was accomplished by using the quantification of TMT reporter ions.

### GO and KEGG analysis

Firstly, the ratio of NBP/MPP + proteins was normalized to rectify the unequal protein content. DEPs were analyzed using a two-tailed t-test after log2 transformation. The p-values were adjusted using “BH method” (PD patients versus health donors). The cutoff of *p* < 0.05 from three replicates and Fold Change > 1.5 was used to define DEPs. Gene Ontology (GO) enrichment and Kyoto Encyclopedia of Genes and Genomes (KEGG) pathway analyses for DEPs were constituted by DAVID (https://david.ncifcrf.gov, version 6.8).

### Protein–Protein Interaction (PPI) Analysis

The PPI networks were explored using STRING v.11.5 (https://cn.string-db.org). Cytoscape v3.9.1 software (Cytoscape Consortium, San Diego, USA) was used to visualize the network and CytoHubba (one of the add-on APP of Cytoscape) was selected to explore the hub proteins by MCC method. In addition, MCL clusting with at least 3 inflation parameters was applied to find out the top 3 clusters. These protein clusters were further uploaded to cytoscape to visualize the complex networks.

### Western blot

The total proteins from N2A cells were lysing with 1% SDS. Subsequently, the extracts were separated using SDS-PAGE on 10% gel and electro-transferred onto PVDF membranes (Bio-Rad, USA), then blocked with 5% nonfat milk at room temperature. The blots were incubated with the primary antibodies P53 (1:1000, abcam, ab246550) and Bax (1:1000, abcam, ab216494) overnight at 4 ℃. On the second day, the blots were incubated with secondary antibodies (4050–05 or 1031–05, Southern Biotech) for 1 h at room temperature, and visualized on amersham Imager 600. Data was normalized to β-actin and quantified using Image J software (NIH, Bethesda).

### Statistical analysis

The proteomics statistical analyses were performed using R (v3.6.3) in the RStudio environment (v1.0.143). The WB statistical analysis were performed using SPSS 26.0 statistical software. Protein expression levels were clustered according to the protein Z-score before visualization using a heatmap. All data were presented as mean ± SEM, and analyzed by one-way analysis of variance (ANOVA) followed by Tukey-HSD test for intergroup differences. A value of *P* < 0.05 was considered statistical significance.

## Result

### The protective role of NBP in reducing mpp + -induced cytotoxicity

**Fig. 1:**
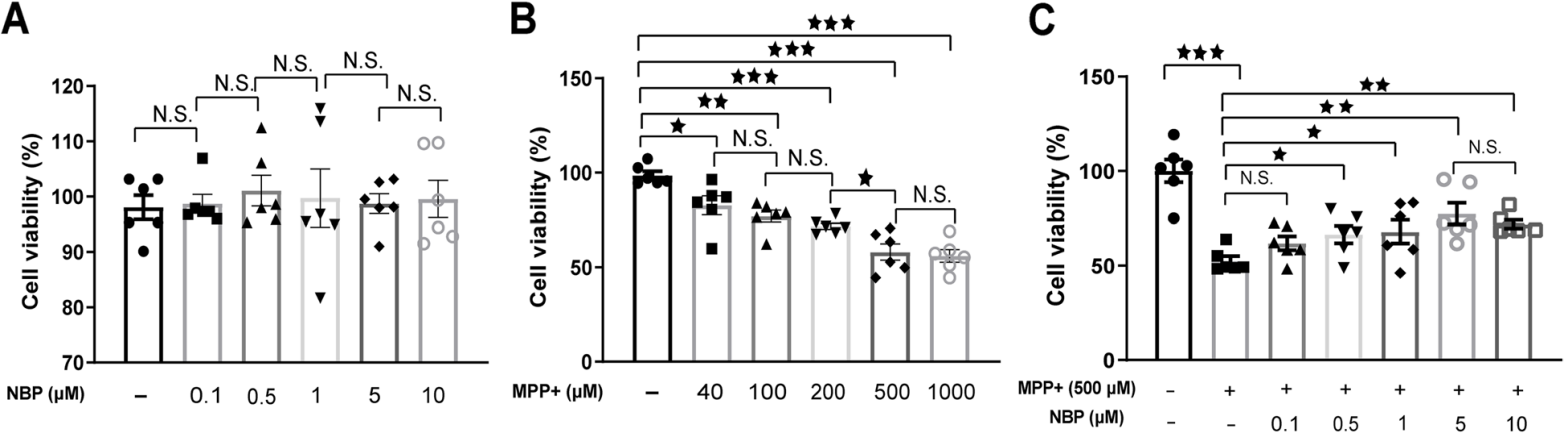
Effect of NBP on MPP + -induced cytotoxicity of N2A cells. (**A**) Cells were stimulated with NBP at a concentration of 0, 0.1, 0.5, 1, 5 or 10 μM for 24 h, and the cell viability was determined by MTT assay. (**B**) Different concentrations of MPP^+^ (0, 40, 100, 250, 500 and 1000 μM) was added to co-cultured with N2A cells for 24 h, and the cell viability was assayed with MTT. (**C**) Cells were pretreated with different concentrations of NBP (0, 0.1, 0.5, 1, 5 or 10 μM) for 24 h, and then these cells were subjected to MPP^+^ (500 μM) insults

The viability of N2A cells in different treatment groups was first determined using MTT assays (Fig. 1). As indicated in Fig. 1A, the different concentration of NBP treatment alone had no significant effect on N2A cells (*P* > 0.05). In contrast, 24 h mpp + treatment induced irreversible cytotoxicity in N2A cells in a dose-dependent manner (Fig. 1B). Our result showed 500 uM mpp + resulted in almost 50% cell death compared with control group (58.1% ± 0.04 vs 98.6% ± 0.02, *P* = 0.03). Though 1000 uM mpp + treatment also decreased cell survival rate to 56.1% ± 0.03, the difference compared with 500 uM mpp + treatment group was not significant. The protective effect of NBP pretreatment on 500 uM mpp + -induced N2A cells was also detected and the result displayed in Fig. 1C showed that 0.1, 0.5, 1, 5, 10 uM NBP significantly increased survival rate after mpp + treatment, especially at 5 uM (77.6% ± 0.06, *P* = 0.01).

### The protein profile of mpp + -induced N2A Cells after NBP treatment

**Fig. 2:**
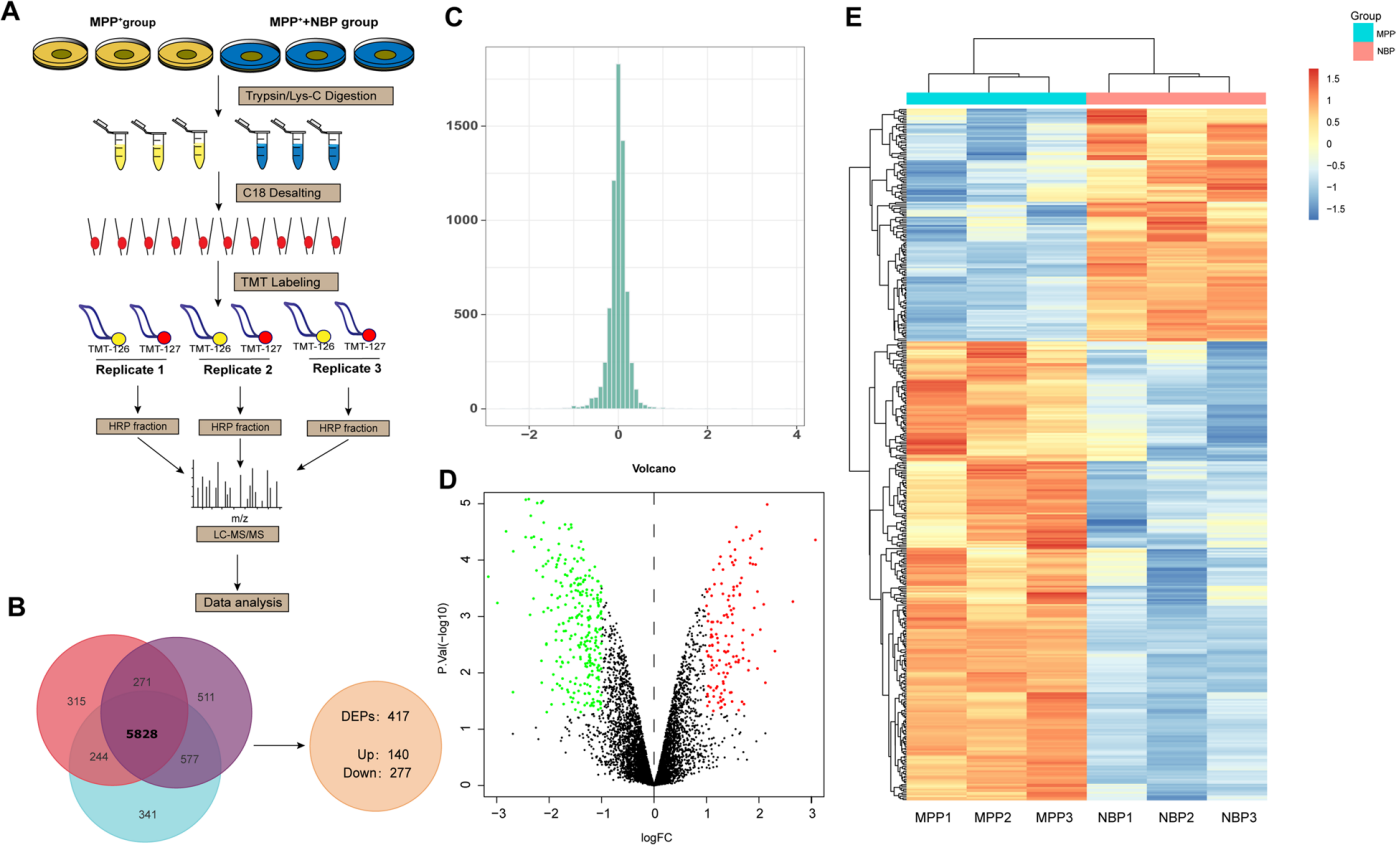
The quantitative proteomics analysis of the protective effect of Dl-3-n-Butylphthalide (NBP). (**A**) The proteomics workflow for the current study. (**B**) In N2a cells which were stimulated with mpp^+^ (500 μM) in the absence or presence of NBP (40 μM) for 24 h, a total of 5829 proteins were identified in three replicates. Using the cut off of |fold-change|> 1.5 and p value < 0.05 to determine DEPs, we qualified 417 proteins and determined 277 increased and 140 decreased. (**C**) Overall distribution of the ratios of 5829 proteins in the quantitative proteomics with three replicates. (**D**) Volcano plot of mpp + -treated group and NBP group. The volcanic map was drawn using two factors, the fold change (Log2) between the two groups of samples and the *p* value (− Log10) obtained by the t-test, to show the significant difference in the data of the two groups of samples. The red dots in the figure are proteins that are significantly differently up-regulated, and the green dots are proteins that down-regulated. Gray dots indicate non-significantly DEPs. (**E**) Heat map of DEPs ratio, each row represents a protein, each column represents the ratio of a sample to a reference sample, and the ratio takes the value of log2

The workflow of the present quantitive proteomic analysis was demonstrated in Fig. 2A. We labeled mpp + -treated samples and NBP pretreated samples with TMT m/z = 126 N, 126C in three independent experiments. In experiment 1, 7273 proteins were identified, of which 6970 (95.83%) were quantified. In experiment 2 and 3, 6955 and 7483 proteins were identified, while 6657 (95.72%) and 7167 (95.79%) proteins were quantified, respectively. In conclusion, a total of 5828 proteins were quantified in the three replicates (Fig. 2B). Using log|fold-change|> 0.58 and *P* < 0.05 as the cutoff, 417 proteins were determined as DEPs. Among the 417 DEPs, 140 were upregulated and 277 were downregulated in NBP pretreatment group compared to control group (Fig. 2B). The complete list of DEPs is revealed in Table 1. The distribution of the log2 expression ratios (NBP + mpp + /mpp +) of the quantified proteins was roughly normal (Fig. 2C). Volcano plot showed DEPs between mpp + -treated group and NBP pretreatment group (Fig. 2D). In addition, the DEPs were also visualized by a heatmap in Fig. 2E.

**Table 1:** The identified DEPs between mpp + group and NBP treatment group using TMT approach

Accession	Gene	Description	P-value
S4R294	Prrc2c-1	Protein PRRC2C	0.029489
S4R270	Bin2	Bridging integrator 2	9.46E-05
Q9Z2Q5	Mrpl40	\39S ribosomal protein L40, mitochondrial \""	0.042355
Q9Z247	Fkbp9	Peptidyl-prolyl cis–trans isomerase FKBP9	0.038663
Q9Z1W9	Stk39	STE20/SPS1-related proline-alanine-rich protein kinase	0.018738
Q9Z1G3	Atp6v1c1	V-type proton ATPase subunit C 1	0.000509
Q9Z0P4	Palm	Paralemmin-1	0.005991
Q9Z0J0	Npc2	NPC intracellular cholesterol transporter 2	6.67E-05
Q9Z0H4-9	Celf2	Isoform 9 of CUGBP Elav-like family member 2	0.000429
Q9WUU7	Ctsz	Cathepsin Z	9.99E-05
Q9WUQ5	Cxcl14	C-X-C motif chemokine 14	0.003162
Q9WU81-2	Slc37a2	Isoform 2 of Glucose-6-phosphate exchanger SLC37A2	0.012391
Q9WTQ5	Akap12	A-kinase anchor protein 12	0.000114
Q9R112	Sqor	\Sulfide:quinone oxidoreductase, mitochondrial \""	0.012363
Q9R0Q6	Arpc1a	Actin-related protein 2/3 complex subunit 1A	0.002284
Q9R0P9	Uchl1	Ubiquitin carboxyl-terminal hydrolase isozyme L1	1.03E-05
Q9QZS2	Rnf4	E3 ubiquitin-protein ligase RNF4	0.002407
Q9QZK7	Dok3	Docking protein 3	0.008101
Q9QZ03	Slc39a1	Zinc transporter ZIP1	0.001082
Q9QXX4	Slc25a13	Calcium-binding mitochondrial carrier protein Aralar2	0.000237
Q9QXW9	Slc7a8	Large neutral amino acids transporter small subunit 2	0.021672
Q9QXS6	Dbn1	Drebrin	0.005062
Q9JMG7-2	Hdgfl3	Isoform 2 of Hepatoma-derived growth factor-related protein 3	0.005934
Q9JMB0	Gkap1	G kinase-anchoring protein 1	0.02322
Q9JM90	Stap1	Signal-transducing adaptor protein 1	0.001898
Q9JLZ6	Hic2	Hypermethylated in cancer 2 protein	0.000198
Q9JK92	Hspb8	Heat shock protein beta-8	0.000655
Q9JJV2	Pfn2	Profilin-2	0.010189
Q9JJ66	Cdc20	Cell division cycle protein 20 homolog	0.03637
Q9JHL0	Lat2	Linker for activation of T-cells family member 2	0.001819
Q9JHK5	Plek	Pleckstrin	1.63E-05
Q9JHG7	Pik3cg	\Phosphatidylinositol 4,5-bisphosphate 3-kinase catalytic subunit gamma isoform \""	0.011177
Q9JHF7	Hpgds	Hematopoietic prostaglandin D synthase	0.035881
Q9JHF5	Tcirg1	V-type proton ATPase subunit a	0.000208
Q9ESY9	Ifi30	Gamma-interferon-inducible lysosomal thiol reductase	0.000443
Q9ESX2	Sp6	Transcription factor Sp6	0.000545
Q9ES52	Inpp5d	\Phosphatidylinositol 3,4,5-trisphosphate 5-phosphatase 1 \""	0.000383
Q9EQP2	Ehd4	EH domain-containing protein 4	0.002416
Q9EQI8	Mrpl46	\39S ribosomal protein L46, mitochondrial \""	0.033823
Q9EQF6	Dpysl5	Dihydropyrimidinase-related protein 5	0.026023
Q9EQ32	Pik3ap1	Phosphoinositide 3-kinase adapter protein 1	6.46E-05
Q9EPN1	Nbea	Neurobeachin	0.002423
Q9EPC1	Parva	Alpha-parvin	0.017161
Q9DCT8	Crip2	Cysteine-rich protein 2	0.004128
Q9DCJ5	Ndufa8	NADH dehydrogenase [ubiquinone] 1 alpha subcomplex subunit 8	0.006871
Q9DBJ3	Baiap2l1	Brain-specific angiogenesis inhibitor 1-associated protein 2-like protein 1	0.006405
Q9DBG5	Plin3	Perilipin-3	0.000782
Q9DBC7	Prkar1a	cAMP-dependent protein kinase type I-alpha regulatory subunit	0.000148
Q9DB94	Wdr53	WD repeat-containing protein 53	0.00139
Q9DAW9	Cnn3	Calponin-3	0.008382
Q9D8X0	Manbal	Protein MANBAL	6.51E-05
Q9D8T2	Gsdmdc1	Gasdermin-D	0.000191
Q9D8K8	Slc25a39	Solute carrier family 25 member 39	0.022448
Q9D711	Pir	Pirin	0.001711
Q9D6V8	Paip2	Polyadenylate-binding protein-interacting protein 2	0.007452
Q9D6K8	Fundc2	FUN14 domain-containing protein 2	0.006128
Q9D517	Agpat3	1-acyl-sn-glycerol-3-phosphate acyltransferase gamma	0.004153
Q9D4Y3	Rhox2a	Reproductive homeobox 2A	0.006143
Q9D1X0	Nol3	Nucleolar protein 3	0.009009
Q9D1M7	Fkbp11	Peptidyl-prolyl cis–trans isomerase FKBP11	0.001442
Q9D1C1	Ube2c	Ubiquitin-conjugating enzyme E2 C	0.00022
Q9D1A2	Cndp2	Cytosolic non-specific dipeptidase	0.000898
Q9D154	Serpinb1a	Leukocyte elastase inhibitor A	9.01E-06
Q9D0S9	Hint2	\Histidine triad nucleotide-binding protein 2, mitochondrial \""	0.032273
Q9D0M2	Cdca7	Cell division cycle-associated protein 7	0.049852
Q9D0A3	Arpin	Arpin	0.001291
Q9CZS1	Aldh1b1	\Aldehyde dehydrogenase X, mitochondrial \""	0.001256
Q9CZC8	Scrn1	Secernin-1	0.027164
Q9CYL5	Glipr2	Golgi-associated plant pathogenesis-related protein 1	0.002113
Q9CY64	Blvra	Biliverdin reductase A	0.000413
Q9CY50	Ssr1	Translocon-associated protein subunit alpha	0.003753
Q9CXE7	Tmed5	Transmembrane emp24 domain-containing protein 5	0.038331
Q9CXC3	Mgme1	Mitochondrial genome maintenance exonuclease 1	0.001646
Q9CXA2	L3hypdh	Trans-L-3-hydroxyproline dehydratase	0.004158
Q9CWU4	1	UPF0690 protein C1orf52 homolog	0.02008
Q9CWS0	Ddah1	\N(G),N(G)-dimethylarginine dimethylaminohydrolase 1 \""	0.006029
Q9CVD2	Atxn3	Ataxin-3	0.007639
Q9CS42	Prps2	Ribose-phosphate pyrophosphokinase 2	0.002208
Q9CRB6	Tppp3	Tubulin polymerization-promoting protein family member 3	0.000706
Q9CR59	Gadd45gip1	Growth arrest and DNA damage-inducible proteins-interacting protein 1	0.006093
Q9CR51	Atp6v1g1	V-type proton ATPase subunit G 1	0.002233
Q9CQX4	Pclaf	PCNA-associated factor	0.02267
Q9CQN7	Mrpl41	\39S ribosomal protein L41, mitochondrial \""	0.008442
Q9CQ91	Ndufa3	NADH dehydrogenase [ubiquinone] 1 alpha subcomplex subunit 3	0.02098
Q9CQ75	Ndufa2	NADH dehydrogenase [ubiquinone] 1 alpha subcomplex subunit 2	0.007796
Q9CQ62	Decr1	\2,4-dienoyl-CoA reductase, mitochondrial \""	0.01819
Q9CQ40	Mrpl49	\39S ribosomal protein L49, mitochondrial \""	0.001931
Q9CPW3	Mrpl54	\39S ribosomal protein L54, mitochondrial \""	0.009102
Q99PA7	4930550L24Rik	MCG117379	0.000432
Q99P91	Gpnmb	Transmembrane glycoprotein NMB	0.000482
Q99N84	Mrps18b	\28S ribosomal protein S18b, mitochondrial \""	0.003021
Q99N69	Lpxn	Leupaxin	0.015689
Q99LY9	Ndufs5	NADH dehydrogenase [ubiquinone] iron-sulfur protein 5	0.00021
Q99LS3	Psph	Phosphoserine phosphatase	0.000429
Q99LD8	Ddah2	\N(G),N(G)-dimethylarginine dimethylaminohydrolase 2 \""	0.000427
Q99LB4	Capg	\Capping protein (Actin filament), gelsolin-like \""	8.47E-06
Q99JP7	Ggt7	Glutathione hydrolase 7	0.006859
Q921W7	Tes	Testin	0.000473
Q921H8	Acaa1a	\3-ketoacyl-CoA thiolase A, peroxisomal \""	9.24E-05
Q91Z31-2	Ptbp2	Isoform 2 of Polypyrimidine tract-binding protein 2	0.004914
Q91YI0	Asl	Argininosuccinate lyase	0.000268
Q91XV3	Basp1	Brain acid soluble protein 1	0.000964
Q91XC8	Dap	Death-associated protein 1	0.018931
Q91XA2	Golm1	Golgi membrane protein 1	0.003959
Q91WU5	As3mt	Arsenite methyltransferase	8.56E-05
Q91W43	Gldc	\Glycine dehydrogenase (decarboxylating), mitochondrial \""	0.000722
Q91VW3	Sh3bgrl3	SH3 domain-binding glutamic acid-rich-like protein 3	9.31E-05
Q91VT4	Cbr4	Carbonyl reductase family member 4	0.036072
Q8VI93	Oas3	2'-5'-oligoadenylate synthase 3	0.010719
Q8VHX6	Flnc	Filamin-C	0.00051
Q8VEA4	Chchd4	Mitochondrial intermembrane space import and assembly protein 40	0.002081
Q8VD46	Asz1	\Ankyrin repeat, SAM and basic leucine zipper domain-containing protein 1 \""	0.032754
Q8VCN5	Cth	Cystathionine gamma-lyase	9.08E-05
Q8R1G6	Pdlim2	PDZ and LIM domain protein 2	0.000344
Q8R1F1	Fam129b	Niban-like protein 1	0.000115
Q8R191	Syngr3	Synaptogyrin-3	0.000281
Q8R035	Mrpl58	\Peptidyl-tRNA hydrolase ICT1, mitochondrial \""	0.001704
Q8QZR5	Gpt	Alanine aminotransferase 1	0.000362
Q8K4Q8	Colec12	Collectin-12	0.002759
Q8K354	Cbr3	Carbonyl reductase [NADPH] 3	0.020264
Q8K352	Sash3	SAM and SH3 domain-containing protein 3	0.006847
Q8K1X4	Nckap1l	Nck-associated protein 1-like	0.009334
Q8K1I7	Wipf1	WAS/WASL-interacting protein family member 1	0.000154
Q8K1B8	Fermt3	Fermitin family homolog 3	0.003138
Q8K124	Plekho2	Pleckstrin homology domain-containing family O member 2	0.016236
Q8K0U4	Hspa12a	Heat shock 70 kDa protein 12A	0.008774
Q8K0C9	Gmds	\GDP-mannose 4,6 dehydratase \""	0.004532
Q8K078	Slco4a1	Solute carrier organic anion transporter family member 4A1	0.000475
Q8CIH5	Plcg2	\1-phosphatidylinositol 4,5-bisphosphate phosphodiesterase gamma-2 \""	0.000269
Q8CIB5	Fermt2	Fermitin family homolog 2	0.033909
Q8CGP0	Hist3h2bb	Histone H2B type 3-B	0.00061
Q8CGN4	Bcor	BCL-6 corepressor	0.007849
Q8CG29	Myo1f	Myosin IF	0.004777
Q8CG03	Pde5a	\cGMP-specific 3',5'-cyclic phosphodiesterase \""	0.001232
Q8CFT3	Ngfr	\Nerve growth factor receptor (TNFR superfamily, member 16) \""	0.003294
Q8CBC7	Ftsj1	Putative tRNA (cytidine(32)/guanosine(34)-2'-O)-methyltransferase	0.006536
Q8C845	Efhd2	EF-hand domain-containing protein D2	0.000654
Q8C3J5	Dock2	Dedicator of cytokinesis protein 2	0.0228
Q8BYW1	Arhgap25	Rho GTPase-activating protein 25	0.01731
Q8BU85	Msrb3	\Methionine-R-sulfoxide reductase B3, mitochondrial \""	0.001027
Q8BPU7	Elmo1	Engulfment and cell motility protein 1	0.000208
Q8BLF1	Nceh1	Neutral cholesterol ester hydrolase 1	0.00098
Q8BHC1	Rab39b	Ras-related protein Rab-39B	0.002247
Q8BGZ6	Gla	Alpha-galactosidase	0.010911
Q8BGQ7	Aars	\Alanine–tRNA ligase, cytoplasmic \""	8.69E-05
Q8BGB5	Limd2	LIM domain-containing protein 2	0.000117
Q8BG16	Slc6a15	Sodium-dependent neutral amino acid transporter B(0)AT2	0.001698
Q8BFW6	Entpd3	Ectonucleoside triphosphate diphosphohydrolase 3	0.0032
Q80ZM5	H1fx	\H1 histone family, member X \""	6.28E-05
Q80Y14	Glrx5	\Glutaredoxin-related protein 5, mitochondrial \""	0.001642
Q80TY0	Fnbp1	Formin-binding protein 1	0.000657
Q80TB8	Vat1l	Synaptic vesicle membrane protein VAT-1 homolog-like	3.66E-05
Q7TPM6	Fsd1	Fibronectin type III and SPRY domain-containing protein 1	0.0003
Q76LS9	Mindy1	Ubiquitin carboxyl-terminal hydrolase MINDY-1	0.000415
Q71FD7	Fblim1	Filamin-binding LIM protein 1	0.003736
Q6ZQ73	Cand2	Cullin-associated NEDD8-dissociated protein 2	0.020611
Q6XLQ8	Calu	Calumenin	0.000397
Q6RUT7	Ccsmst1	Protein CCSMST1	0.002908
Q6PGB6-4	Naa50	Isoform 4 of N-alpha-acetyltransferase 50	0.003549
Q6P6I8	Sirpa	Signal-regulatory protein alpha	0.005252
Q6NXJ0	Wwc2	Protein WWC2	0.00402
Q6NSP9	Hmga2	High mobility group protein HMGI-C	0.006245
Q6IRU5-2	Cltb	Isoform 2 of Clathrin light chain B	0.00267
Q6DID7	Wls	Protein wntless homolog	0.014365
Q64364	Cdkn2a	Tumor suppressor ARF	0.002669
Q64133	Maoa	Amine oxidase [flavin-containing] A	0.002698
Q63918	Cavin2	Caveolae-associated protein 2	0.000419
Q62433	Ndrg1	Protein NDRG1	0.012657
Q62261	Sptbn1	\Spectrin beta chain, non-erythrocytic 1 \""	4.75E-05
Q61792	Lasp1	LIM and SH3 domain protein 1	0.001071
Q61699	Hsph1	Heat shock protein 105 kDa	0.000327
Q61599	Arhgdib	Rho GDP-dissociation inhibitor 2	0.000289
Q61576	Fkbp10	Peptidyl-prolyl cis–trans isomerase FKBP10	0.000962
Q61553	Fscn1	Fascin	0.000775
Q61490	Alcam	CD166 antigen	0.000237
Q61469-2	Plpp1	Isoform 2 of Phospholipid phosphatase 1	0.002332
Q61469	Plpp1	Phospholipid phosphatase 1	0.003157
Q61462	Cyba	Cytochrome b-245 light chain	0.014978
Q61337	Bad	Bcl2-associated agonist of cell death	0.00141
Q61263	Soat1	Sterol O-acyltransferase 1	0.000342
Q61233	Lcp1	Plastin-2	9.66E-06
Q61214	Dyrk1a	Dual specificity tyrosine-phosphorylation-regulated kinase 1A	0.001767
Q61152	Ptpn18	Tyrosine-protein phosphatase non-receptor type 18	0.000197
Q61140	Bcar1	Breast cancer anti-estrogen resistance protein 1	0.04185
Q61024	Asns	Asparagine synthetase [glutamine-hydrolyzing]	0.000261
Q60865	Caprin1	Caprin-1	0.00169
Q60598	Cttn	Src substrate cortactin	4.45E-05
Q5SXY1	Specc1	Cytospin-B	0.000352
Q5SX75	P4ha2	\Procollagen-proline, 2-oxoglutarate 4-dioxygenase (Proline 4-hydroxylase), alpha II polypeptide, isoform CRA_f \""	0.000207
Q5SSZ5	Tns3	Tensin-3	0.000413
Q5SSL4-2	Abr	Isoform 2 of Active breakpoint cluster region-related protein	0.000541
Q5SF07	Igf2bp2	Insulin-like growth factor 2 mRNA-binding protein 2	0.003528
Q5ISE2	Zfp36l3	mRNA decay activator protein ZFP36L3	0.007734
Q3V460	Smim26	\Gene model 561, (NCBI) \""	0.001804
Q3UZ39	Lrrfip1	Leucine-rich repeat flightless-interacting protein 1	0.00016
Q3UW53	Fam129a	Protein Niban	0.000403
Q3UND0	Skap2	Src kinase-associated phosphoprotein 2	0.002957
Q3ULW8	Parp3	Protein mono-ADP-ribosyltransferase PARP3	0.032281
Q3UKW2	Calm1	Calmodulin-1	0.00326
Q3UKU1	Ell2	RNA polymerase II elongation factor ELL2	6.98E-05
Q3UH59	Myh10	Myosin-10	0.000122
Q3U9N4	Grn	Granulins	0.007805
Q3U816	Htatip2	Oxidoreductase HTATIP2	0.006365
Q3U6Q4	Pik3r6	Phosphoinositide 3-kinase regulatory subunit 6	0.045005
Q3U1Z5	Gpsm3	G-protein-signaling modulator 3	0.000128
Q3U125	Fam213a	\Family with sequence similarity 213, member A \""	0.013245
Q3TW96	Uap1l1	UDP-N-acetylhexosamine pyrophosphorylase-like protein 1	0.000244
Q3TRM8	Hk3	Hexokinase-3	0.001053
Q3TKR3	Nlrp4c	\NACHT, LRR and PYD domains-containing protein 4C \""	0.004146
Q3TH01	H2-K1	\H-2 class I histocompatibility antigen, K-K alpha chain \""	0.000264
Q3TGW2	Eepd1	Endonuclease/exonuclease/phosphatase family domain-containing protein 1	0.027175
Q14B01	Rnf113a2	Ring finger protein 113A2	0.000267
Q0PD20	Rab34	Rab34	0.023811
Q09143	Slc7a1	High affinity cationic amino acid transporter 1	0.032001
Q08509	Eps8	Epidermal growth factor receptor kinase substrate 8	0.006738
Q07813	Bax	Apoptosis regulator BAX	0.018148
Q05915	Gch1	GTP cyclohydrolase 1	0.007424
Q05816	Fabp5	Fatty acid-binding protein 5	0.000335
Q05186	Rcn1	Reticulocalbin-1	0.0001
Q05144	Rac2	Ras-related C3 botulinum toxin substrate 2	0.001842
Q04447	Ckb	Creatine kinase B-type	0.001009
Q01965	Ly9	T-lymphocyte surface antigen Ly-9	0.022723
Q01320	Top2a	DNA topoisomerase 2-alpha	0.000116
Q00651	Itga4	Integrin alpha-4	0.003369
Q00519	Xdh	Xanthine dehydrogenase/oxidase	0.005239
P97863	Nfib	Nuclear factor 1 B-type	0.004333
P97821	Ctsc	Dipeptidyl peptidase 1	0.005292
P97449	Anpep	Aminopeptidase N	0.000102
P97370	Atp1b3	Sodium/potassium-transporting ATPase subunit beta-3	0.000624
P97369	Ncf4	Neutrophil cytosol factor 4	0.00195
P97363	Sptlc2	Serine palmitoyltransferase 2	0.004559
P84102	Serf2	Small EDRK-rich factor 2	0.000133
P70444	Bid	BH3-interacting domain death agonist	0.006639
P70315	Was	Wiskott-Aldrich syndrome protein homolog	0.024818
P70290	Mpp1	55 kDa erythrocyte membrane protein	0.000105
P63082	Atp6v0c	V-type proton ATPase 16 kDa proteolipid subunit	0.023528
P62965	Crabp1	Cellular retinoic acid-binding protein 1	0.000361
P62631	Eef1a2	Elongation factor 1-alpha 2	0.00012
P61961	Ufm1	Ubiquitin-fold modifier 1	0.007187
P60762-2	Morf4l1	Isoform 2 of Mortality factor 4-like protein 1	0.000971
P58681	Tlr7	Toll-like receptor 7	0.038526
P57759	Erp29	Endoplasmic reticulum resident protein 29	0.000502
P57722	Pcbp3	Poly(rC)-binding protein 3	0.00388
P56873	Sssca1	Sjoegren syndrome/scleroderma autoantigen 1 homolog	0.000467
P56391	Cox6b1	Cytochrome c oxidase subunit 6B1	0.00151
P56375	Acyp2	Acylphosphatase-2	0.000927
P55302	Lrpap1	Alpha-2-macroglobulin receptor-associated protein	0.000424
P55097	Ctsk	Cathepsin K	0.000414
P50543	S100a11	Protein S100-A11	3.70E-05
P50396	Gdi1	Rab GDP dissociation inhibitor alpha	2.60E-05
P49710	Hcls1	Hematopoietic lineage cell-specific protein	8.07E-05
P49138	Mapkapk2	MAP kinase-activated protein kinase 2	0.045037
P48774	Gstm5	Glutathione S-transferase Mu 5	0.002214
P48722	Hspa4l	Heat shock 70 kDa protein 4L	0.003301
P48678	Lmna	Prelamin-A/C	0.001037
P48036	Anxa5	Annexin A5	0.000223
P48025	Syk	Tyrosine-protein kinase SYK	0.00388
P47738	Aldh2	\Aldehyde dehydrogenase, mitochondrial \""	0.00029
P47713	Pla2g4a	Cytosolic phospholipase A2	2.33E-05
P46656	Fdx1	\Adrenodoxin, mitochondrial \""	0.033399
P46414	Cdkn1b	Cyclin-dependent kinase inhibitor 1B	0.001209
P45952	Acadm	\Medium-chain specific acyl-CoA dehydrogenase, mitochondrial \""	0.000374
P43276	Hist1h1b	Histone H1.5	0.026301
P43275	Hist1h1a	Histone H1.1	4.39E-05
P43135	Nr2f2	COUP transcription factor 2	0.005166
P40240	Cd9	CD9 antigen	0.00748
P40124	Cap1	Adenylyl cyclase-associated protein 1	2.65E-05
P37913	Lig1	DNA ligase 1	0.000255
P35991	Btk	Tyrosine-protein kinase BTK	0.001154
P35505	Fah	Fumarylacetoacetase	0.005521
P30412	Ppic	Peptidyl-prolyl cis–trans isomerase C	0.000181
P30282	Ccnd3	G1/S-specific cyclin-D3	0.009968
P29351-2	Ptpn6	Isoform 2 of Tyrosine-protein phosphatase non-receptor type 6	2.34E-05
P28738	Kif5c	Kinesin heavy chain isoform 5C	0.035953
P28574	Max	Protein max	0.016467
P27870	Vav1	Proto-oncogene vav	0.003988
P26645	Marcks	Myristoylated alanine-rich C-kinase substrate	0.022253
P26011	Itgb7	Integrin beta-7	0.000253
P24668	M6pr	Cation-dependent mannose-6-phosphate receptor	0.023417
P24472	Gsta4	Glutathione S-transferase A4	0.032647
P24288	Bcat1	\Branched-chain-amino-acid aminotransferase, cytosolic \""	0.000362
P21956	Mfge8	Lactadherin	0.000274
P21550	Eno3	Beta-enolase	0.000237
P20491	Fcer1g	High affinity immunoglobulin epsilon receptor subunit gamma	3.91E-05
P19973	Lsp1	Lymphocyte-specific protein 1	0.000778
P17047-2	Lamp2	Isoform LAMP-2B of Lysosome-associated membrane glycoprotein 2	0.005181
P16546	Sptan1	\Spectrin alpha chain, non-erythrocytic 1 \""	0.00011
P16460	Ass1	Argininosuccinate synthase	2.21E-06
P16110	Lgals3	Galectin-3	5.90E-05
P14901	Hmox1	Heme oxygenase 1	0.001048
P14873	Map1b	Microtubule-associated protein 1B	3.11E-05
P14824	Anxa6	Annexin A6	0.000366
P13020	Gsn	Gelsolin	0.00029
P11928	Oas1a	2'-5'-oligoadenylate synthase 1A	0.00249
P11835	Itgb2	Integrin beta-2	0.001344
P11404	Fabp3	\Fatty acid-binding protein, heart \""	0.000104
P11152	Lpl	Lipoprotein lipase	0.00131
P10922	H1f0	Histone H1.0	4.15E-05
P10852-2	Slc3a2	Isoform 2 of 4F2 cell-surface antigen heavy chain	0.000647
P10605	Ctsb	Cathepsin B	0.000225
P10518	Alad	Delta-aminolevulinic acid dehydratase	0.000903
P10107	Anxa1	Annexin A1	4.86E-06
P0DOV2	Ifi204	Interferon-activable protein 204	0.000694
P0C7L0	Wipf3	WAS/WASL-interacting protein family member 3	0.000337
P0C605	Prkg1	cGMP-dependent protein kinase 1	0.00734
P09581	Csf1r	Macrophage colony-stimulating factor 1 receptor	0.049641
P09528	Fth1	Ferritin heavy chain	0.000414
P08905	Lyz2	Lysozyme C-2	8.27E-06
P08226	Apoe	Apolipoprotein E	0.000234
P08207	S100a10	Protein S100-A10	0.000829
P07356	Anxa2	Annexin A2	0.000359
P07309	Ttr	Transthyretin	0.011883
P06869	Plau	Urokinase-type plasminogen activator	2.71E-05
P06800	Ptprc	Receptor-type tyrosine-protein phosphatase C	0.001072
P06797	Ctsl	Cathepsin L1	0.000278
P04117	Fabp4	\Fatty acid-binding protein, adipocyte \""	4.01E-05
P03975	Iap	IgE-binding protein	0.002911
P03958	Ada	Adenosine deaminase	0.017508
P02802	Mt1	Metallothionein-1	3.09E-05
P02798	Mt2	Metallothionein-2	0.000576
P02340	Tp53	Cellular tumor antigen p53	4.68E-05
P01899	H2-D1	\H-2 class I histocompatibility antigen, D-B alpha chain \""	0.010832
P01887	B2m	Beta-2-microglobulin	0.001055
O89053	Coro1a	Coronin-1A	9.79E-05
O88188	Ly86	Lymphocyte antigen 86	0.000189
O70209	Pdlim3	PDZ and LIM domain protein 3	0.040746
O70145	Ncf2	Neutrophil cytosol factor 2	0.00102
O55003	Bnip3	BCL2/adenovirus E1B 19 kDa protein-interacting protein 3	0.001351
O54926	Siva1	Apoptosis regulatory protein Siva	0.002316
O54879	Hmgb3	High mobility group protein B3	6.00E-05
O54724	Cavin1	Caveolae-associated protein 1	0.007188
O35887	Calu	Calumenin	0.000147
O35874	Slc1a4	Neutral amino acid transporter A	0.029691
O35690	Phox2b	Paired mesoderm homeobox protein 2B	0.000918
O35639	Anxa3	Annexin A3	5.15E-05
O35601	Fyb1	FYN-binding protein 1	0.000189
O35075	Dscr3	Down syndrome critical region protein 3 homolog	0.000377
O09131	Gsto1	Glutathione S-transferase omega-1	2.83E-05
O09047	C3ar1	C3a anaphylatoxin chemotactic receptor	0.003033
O08804	Serpinb6b	NK13	0.000689
O08749	Dld	\Dihydrolipoyl dehydrogenase, mitochondrial \""	0.00057
J3QPG5	Psap	Prosaposin	0.002153
J3QN31	Adssl1	Adenylosuccinate synthetase isozyme 1	9.71E-06
H3BL08	Cers6	Ceramide synthase 6	0.016882
H3BJD6	Ppp1r9a	\Protein phosphatase 1, regulatory subunit 9A \""	0.001305
G5E8L6	Klrg2	Killer cell lectin-like receptor subfamily G member 2	0.005462
G3X9H7	Mtss1	\Metastasis suppressor 1, isoform CRA_e \""	0.006606
G3X8Y8	Tlr2	Toll-like receptor 2	0.028337
G3X8Y3	Naa15	\N-alpha-acetyltransferase 15, NatA auxiliary subunit \""	0.000656
G3X8T3	Ctsa	Carboxypeptidase	0.000151
G3X8S8	Tsen15	MCG14499	0.005902
G3UYX7	Slit2	Slit homolog 2 protein	0.015515
F8WIV2	Serpinb6a	\Serine (or cysteine) peptidase inhibitor, clade B, member 6a \""	5.60E-05
F8WIP8	Spp1	Osteopontin	0.000708
F8WHQ1	Tpd52	Tumor protein D52	0.037531
F8WH69	Ncf1	Neutrophil cytosol factor 1	0.000616
F8WGM5	Stxbp2	Syntaxin-binding protein 2 (Fragment)	0.000862
F8WGF2	Nos1	\Nitric oxide synthase, brain \""	0.010364
F8VQ28	Pxn	Paxillin	0.007338
F7DBB3	Ahnak2	AHNAK nucleoprotein 2 (Fragment)	3.84E-05
F7CVJ5	Ahnak2	AHNAK nucleoprotein 2 (Fragment)	2.16E-05
F6WR04	Ctss	Cathepsin S	0.000153
F6WMJ3	Arhgef6	Rho guanine nucleotide exchange factor 6	0.000142
F6TZU3	Gan	Gigaxonin (Fragment)	0.031505
E9QQ25	Speg	Striated muscle-specific serine/threonine-protein kinase	0.005699
E9QP49	Ehbp1l1	EH domain-binding protein 1-like protein 1	0.034701
E9QMK9	Dglucy	\D-glutamate cyclase, mitochondrial \""	0.037768
E9QLZ9	Enah	Protein enabled homolog	0.005109
E9QA16	Cald1	Caldesmon 1	0.00029
E9QA15	Cald1	Caldesmon 1	0.002066
E9Q7X7	Nrxn2	Neurexin-2	0.020083
E9Q634	Myo1e	Unconventional myosin-Ie	0.000582
E9Q414	Apob	Apolipoprotein B-100	0.014265
E9Q3X0	Mvp	Major vault protein	0.002328
E9Q3L4	Ifi207	Interferon-activated gene 207	0.00012
E9Q3F7	Peg10	Retrotransposon-derived protein PEG10	0.008086
E9PYB0	Ahnak2	AHNAK nucleoprotein 2 (Fragment)	0.000817
E9PWE8	Dpysl3	Dihydropyrimidinase-related protein 3	0.000118
E9PVB7	Satb1	DNA-binding protein SATB	0.006487
D3Z383	Mest	\Mesoderm specific transcript, isoform CRA_a \""	0.005248
D0QMC3	Mndal	Myeloid cell nuclear differentiation antigen-like protein	0.007196
B8QI34	Ppfia2	Liprin-alpha-2	0.014999
B1AX58	Pls3	Plastin-3	0.002622
B1ASZ3	Gk	Glycerol kinase	0.033759
B1AR13	Cisd3	\CDGSH iron-sulfur domain-containing protein 3, mitochondrial \""	0.000256
A7YY80	Epb41l3	130 kDa Protein 4.1B MEF cell isoform	0.001257
A2AUD5	Tpd52l2	Tumor protein D54	0.00548
A2AQ87	Shf	SH2 domain-containing adapter protein F (Fragment)	0.036499
A2APR8	Bub1	Mitotic checkpoint serine/threonine-protein kinase BUB1	0.009011
A2AFI6	Gm364	Transmembrane 9 superfamily member	0.002941
A2A7S8-2	Kiaa1522	Isoform 2 of Uncharacterized protein KIAA1522	0.021972
A2A7P9	Svbp	\Coiled-coil domain containing 23, isoform CRA_c \""	0.031763
A2A7A7	H6pd	GDH/6PGL endoplasmic bifunctional protein	0.015564
A0A2I3BR29	Fam107b	Protein FAM107B	0.012061
A0A338P769	Septin-5	Septin-5	0.011855
A0A286YDF5	Myof	Myoferlin	0.000487
A0A1Y7VM56	Sirt5	\NAD-dependent protein deacylase sirtuin-5, mitochondrial \""	0.015673
A0A1W2P775	Itsn2	Intersectin-2 (Fragment)	0.0213
A0A1W2P6X3	Fmnl1	Formin-like protein 1	0.003337
A0A1B0GSG5	Rnh1	Ribonuclease inhibitor	0.000131
A0A140T8J4	Hebp1	Heme-binding protein 1	0.000499
A0A140T8I6	Epsti1	Epithelial stromal interaction 1 (Breast)	0.032835
A0A140LIZ7	Nhsl1	NHS-like protein 1	0.045976
A0A0R4J2B2	Kctd12	BTB/POZ domain-containing protein KCTD12	0.00608
A0A0R4J1C8	Cd68	Macrosialin	0.034522
A0A0R4J104	Dab2	Disabled homolog 2	4.29E-05
A0A0R4J0S1	Cdc42ep1	Cdc42 effector protein 1	0.047415
A0A0R4J0K5	Cd84	SLAM family member 5	0.001207
A0A0R4J0I9	Lrp1	Low density lipoprotein receptor-related protein 1	0.000814
A0A0R4J0A4	Flt1	Vascular endothelial growth factor receptor 1	9.14E-05
A0A0R4J049	Prmt5	Protein arginine N-methyltransferase 5	0.022053
A0A0N4SW28	Gng12	Guanine nucleotide-binding protein subunit gamma	0.000936
A0A0G2JGX4	Atp1a3	Sodium/potassium-transporting ATPase subunit alpha	0.000912
A0A0G2JEK2	Crip1	Cysteine-rich protein 1	0.002123
A0A087WS96	Sh3bgrl2	SH3 domain-binding glutamic acid-rich-like protein	0.003135
A0A087WQT6	Casp8	Caspase-8	0.002808
A0A087WPF7	Auts2	Autism susceptibility gene 2 protein homolog	0.00174

### Bioinformatics analysis of the identified DEPs

#### GO and KEGG analysis of DEPs

**Fig. 3:**
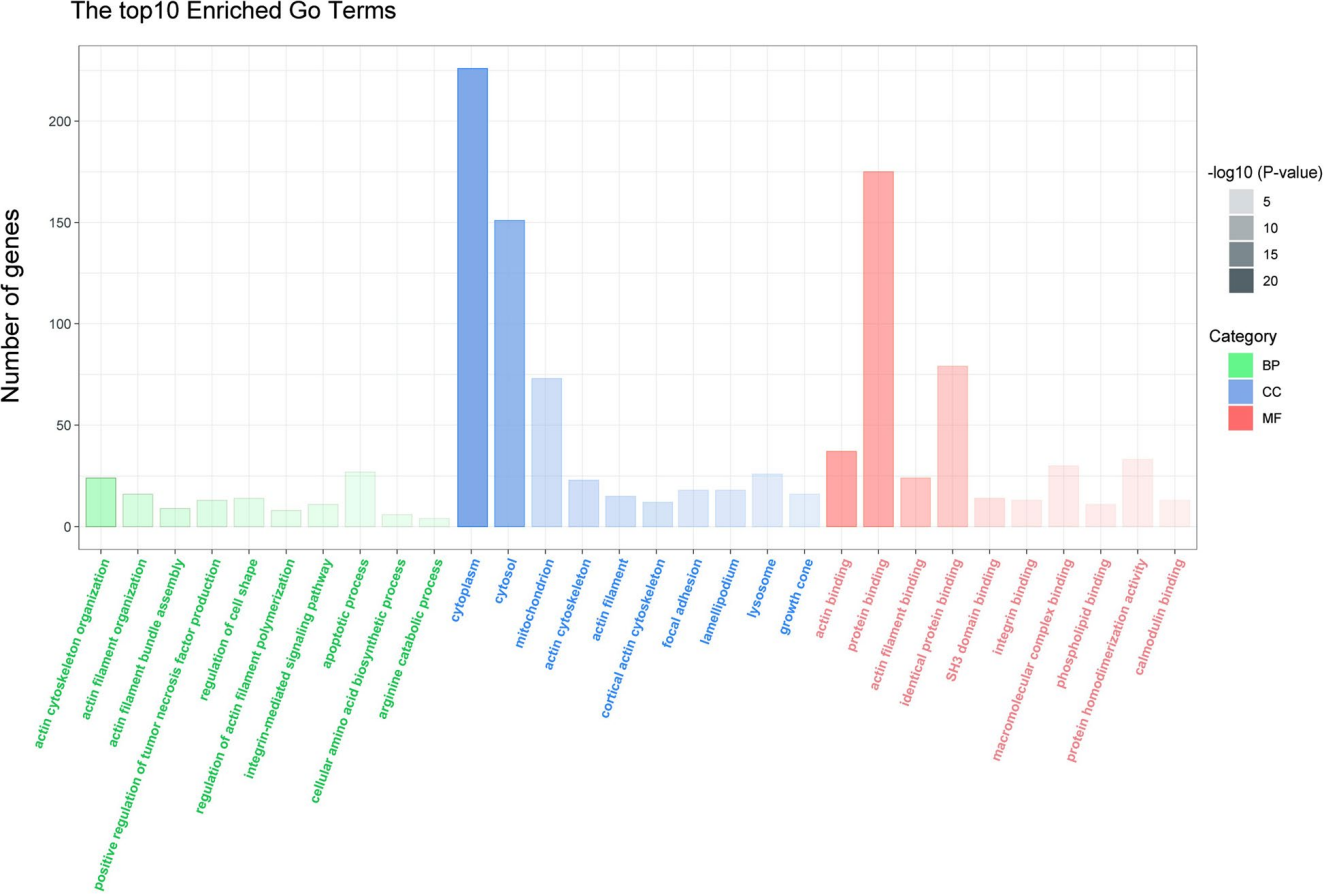
Ontological analysis of the DEPs. Classification of the DEPs based on cellular component (CC), biological process (BP) and molecular function (MF). We listed top 10 enriched GO pathways. The ordinate represents number of differential proteins contained in each classification and the abscissa represents the significantly enriched functional classification and pathway

**Fig. 4:**
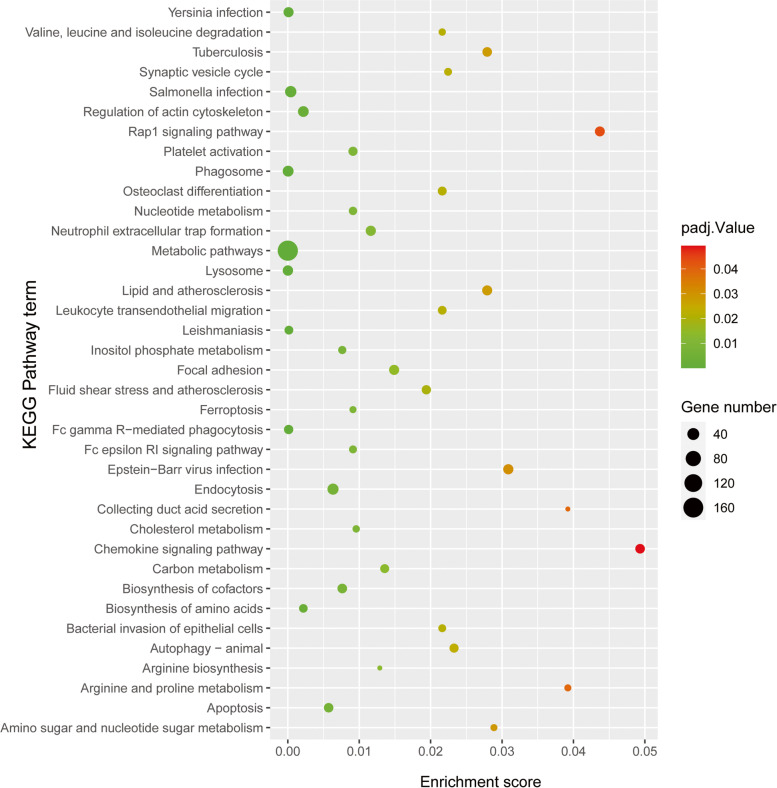
KEGG Pathway Enrichment of DEPs. The X axis is the enrichment fraction, and the Y axis is the KEGG pathway term. Node area was positively correlated with the number of genes expressed, and node color was positively correlated with the enrichment analysis score

To better understand the cellular location, functions and the involved biological pathways of the DEPs, Gene Oncology (GO) analysis were conducted (Fig. 3). According to our analysis, the DEPs were found to be mainly localized to the cytoplasm, cytosol, mitochondrion, actin cytoskeleton and actin filamentactin. The GO-biological processes (BP) analysis revealed that DEPs were related to actin cytoskeleton organization, actin filament organization, actin filament bundle assembly, positive regulation of tumor necrosis factor production, regulation of cell shape and so forth. Regarding to GO-molecular functions (MF), DEPs were biased towards actin binding, protein binding, actin filament binding, identical protein binding, SH3 domain binding and integrin binding. KEGG pathway enrichment analysis was also performed to understand the associated pathways of the DEPs (Fig. 4). The result indicated that lysosome, phagosome, apoptosis, endocytosis, cholesterol metabolism and ferroptosis were the mainly enriched pathways.

### Mapping STRING Protein–Protein Interaction (PPI) Network

**Fig. 5:**
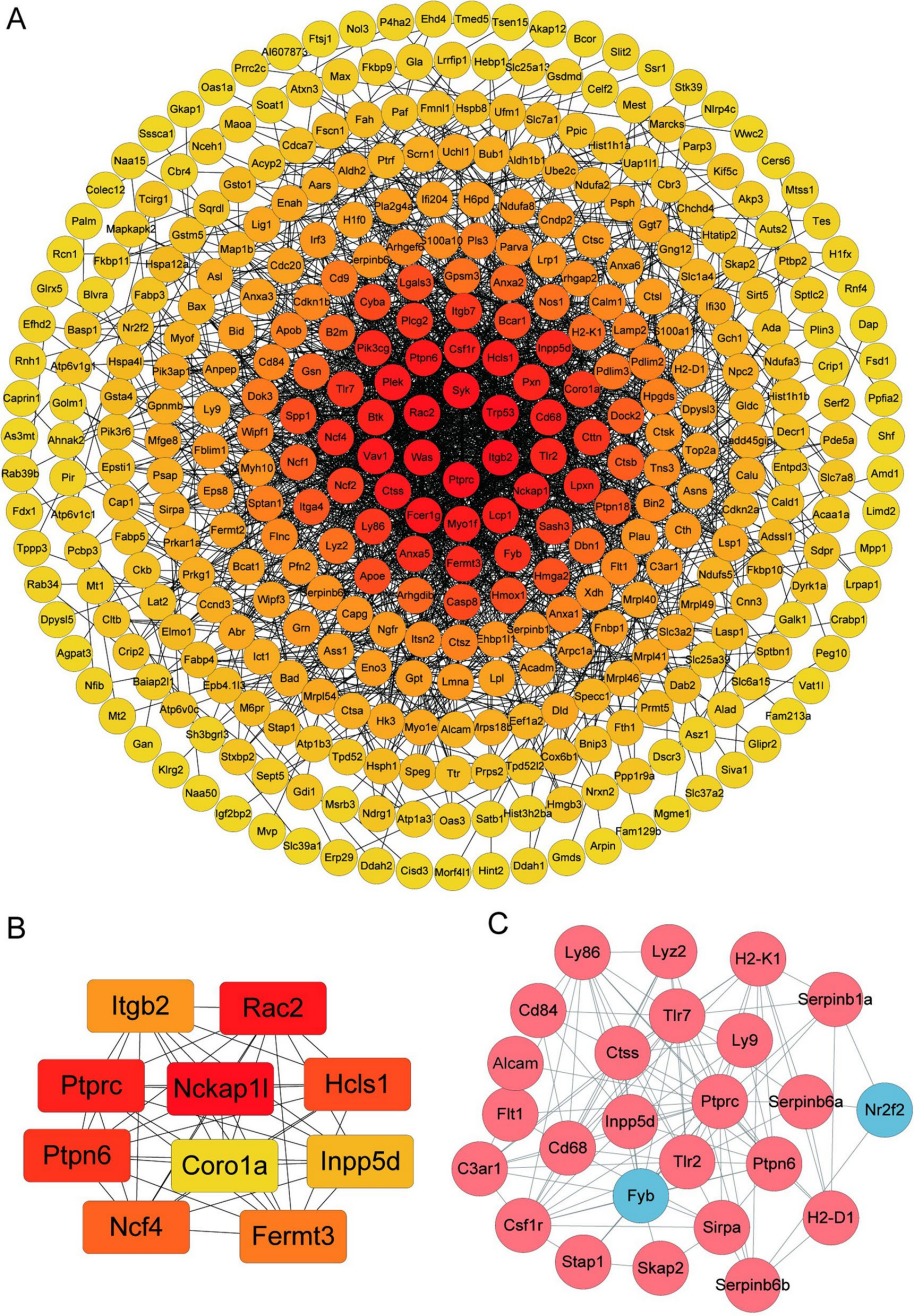
(**A**) Protein–protein interaction network consisting of 417 DEPs (**B**). The color saturation of the edges represents the confidence score of the association between modulated proteins. The top 10 proteins with the highest degree of PPI network connectivity were identified by the MMC method using CytoHubba. (**C**) The differentially regulated cluster I protein network is visualized using Cytoscape 3.8.0. The blue nodes indicate the significantly downregulated proteins and red nodes represent the significantly upregulated proteins (*p* < 0.05)

The DEPs were uploaded to the STRING online tool to identify the protein–protein interactions and the biological significance of the identified proteins. Cytoscape was used to visualized the network. The result demonstrated that the DEPs constructed a complicated interaction network with 412 nodes and 1999 edges. The clustering coefficient was 0.388. As the expected edges number was 902, our result displayed a much higher value than the expected edges. The confident of PPI enrichment was *p* < 1 × 10^−16^. Likewise, the tight PPI network demonstrated in Fig. 5A also suggested the strong interaction between DEPs. Furthermore, screening the DEPs by CytoHubba App using the Maximum Clique Centrality (MCC) method in Cytoscape software, the top 10 hub proteins were recognized, namely, Integrin β2 (ITGB2), Ras-related C3 botulinum toxin substrate 2 (Rac2), Receptor-type tyrosine-protein phosphatase C (Ptprc), Nck-associated protein 1-like (Nckap 11), Hematopoietic lineage cell-specific protein (Hcls1), Isoform 2 of Tyrosine-protein phosphatase non-receptor type 6 (Ptpn6), Coronin-1A (Coro1a), Phosphatidylinositol 3,4,5-trisphosphate 5-phosphatase 1 (Inpp5d), Neutrophil cytosol factor 4 (Ncf4) and Fermitin family homolog 3 (Fermt3)(Fig. 5B).

### Cluster Analysis of DEPs

By using MCL clustering, 48 clusters were generated which contained more than 3 genes. We analyzed the top 3 generated clusters to recognize the therapeutic target and dominant pathway for NBP treatment. In Fig. 5C, there were 25 Proteins in cluster I namely, CD166 antigen (Alcam), C3a anaphylatoxin chemotactic receptor (C3ar1), Macrosialin (Cd68), SLAM family member 5 (Cd84), Macrophage colony-stimulating factor 1 receptor (Csf1r), Cathepsin S (Ctss), Vascular endothelial growth factor receptor 1 (Flt1), FYN-binding protein 1 (Fyb), H-2 class I histocompatibility antigen, D-B alpha chain(H2-D1), H-2 class I histocompatibility antigen, K-K alpha chain (H2-K1), Phosphatidylinositol 3,4,5-trisphosphate 5-phosphatase 1 (Inpp5d), Lymphocyte antigen 86 (Ly86), T-lymphocyte surface antigen Ly-9 (Ly9), Lysozyme C-2 (Lyz2), COUP transcription factor 2 (Nr2f2), Isoform 2 of Tyrosine-protein phosphatase non-receptor type 6 (Ptpn6), Receptor-type tyrosine-protein phosphatase C (Ptprc), Leukocyte elastase inhibitor A (Serpinb1a), Serine peptidase inhibitor (Serpinb6a), NK13 (Serpinb6b), Signal-regulatory protein alpha (Sirpa), Src kinase-associated phosphoprotein 2 (Skap2), Signal-transducing adaptor protein 1 (Stap1), Toll-like receptor 2 (Tlr2), Toll-like receptor 7 (Tlr7). The enrichment KEGG pathways of cluster I proteins were involved Phagosome (FDR value = 0.0097), Cell adhesion molecules (FDR value = 0.0097), Antigen processing and presentation (FDR value = 0.0098) and Natural killer cell mediated cytotoxicity (FDR value = 0.0201).

**Fig. 6:**
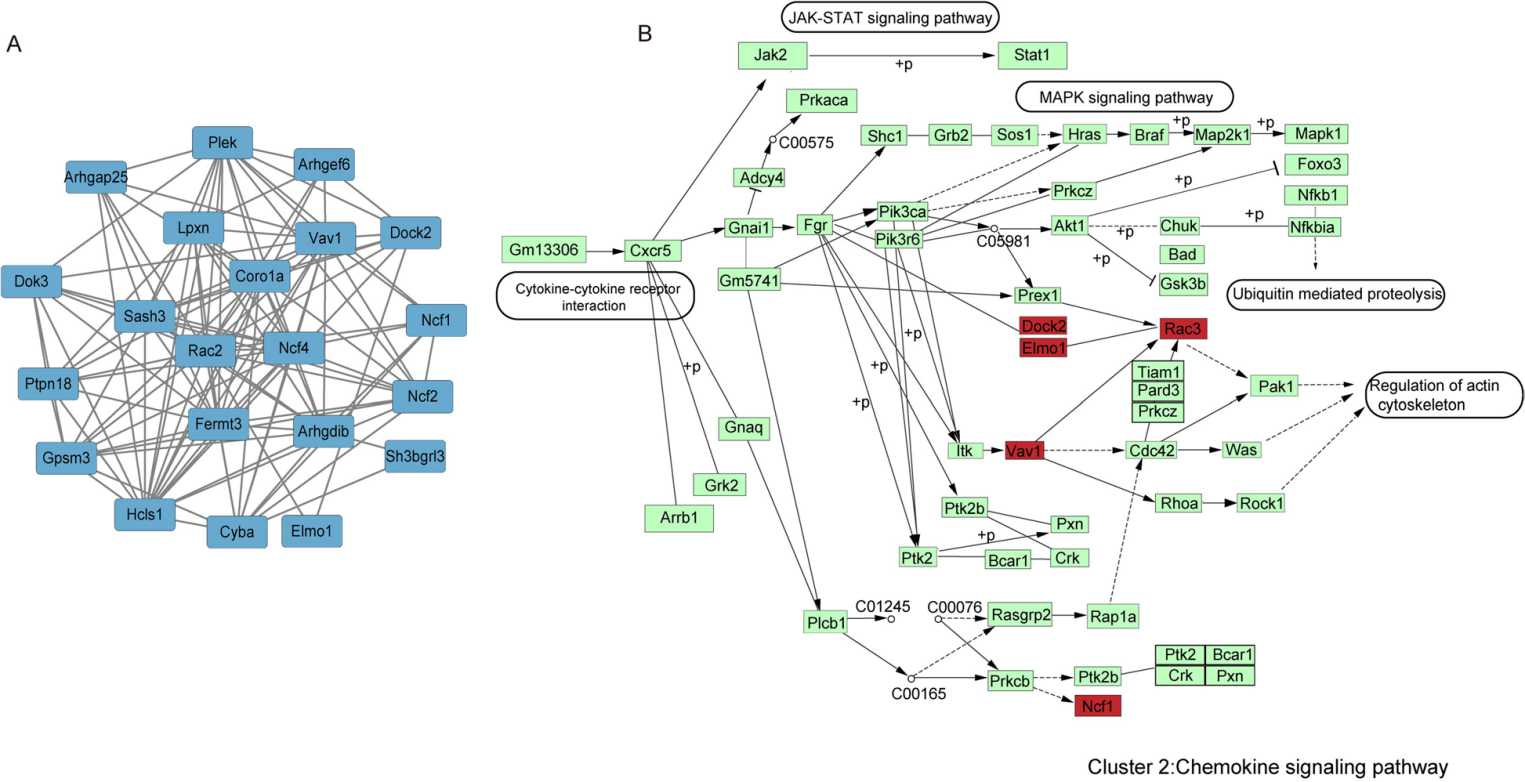
KEGG pathway enrichment analysis of cluster II proteins (**A**). The differentially regulated cluster II protein network in mpp^+^-stimulated N2A cells in response to NBP is visualized using Cytoscape 3.8.0. The blue nodes indicate the significantly downregulated cluster II proteins (*p* < 0.05). (**B**) The position of cluster III proteins in the KEGG “Chemokine signaling pathway” during the NBP treatment are shown in red color

The proteins In cluster II included Rho GTPase-activating protein 25 (Arhgap25), Rho GDP-dissociation inhibitor 2 (Arhgdib), Rho guanine nucleotide exchange factor 6 (Arhgef6), Coronin-1A (Coro1a), Cytochrome b-245 light chain (Cyba), Dedicator of cytokinesis protein 2 (Dock2), Docking protein 3 (Dok3), Engulfment and cell motility protein 1 (Elmo1), Fermitin family homolog 3 (Fermt3), G-protein-signaling modulator 3 (Gpsm3), Hematopoietic lineage cell-specific protein (Hcls1), Leupaxin (Lpxn), Neutrophil cytosol factor 1 (Ncf1), Neutrophil cytosol factor 2 (Ncf2),Neutrophil cytosol factor 4 (Ncf4), Pleckstrin (Plek), Tyrosine-protein phosphatase non-receptor type 18 (Ptpn18), Ras-related C3 botulinum toxin substrate 2 (Rac2), SAM and SH3 domain-containing protein 3 (Sash3), SH3 domain-binding glutamic acid-rich-like protein 3 (Sh3bgrl3), Proto-oncogene vav (Vav1)(Fig. 6A). The KEGG enrichment analysis was also conducted and the result showed that leukocyte transendothelial migration, chemokine signaling pathway and phagosome were the top 3 enriched terms of KEGG pathway. We deliberately explained the proteins involved in chemokine signaling pathway in Fig. 6B.

**Fig. 7:**
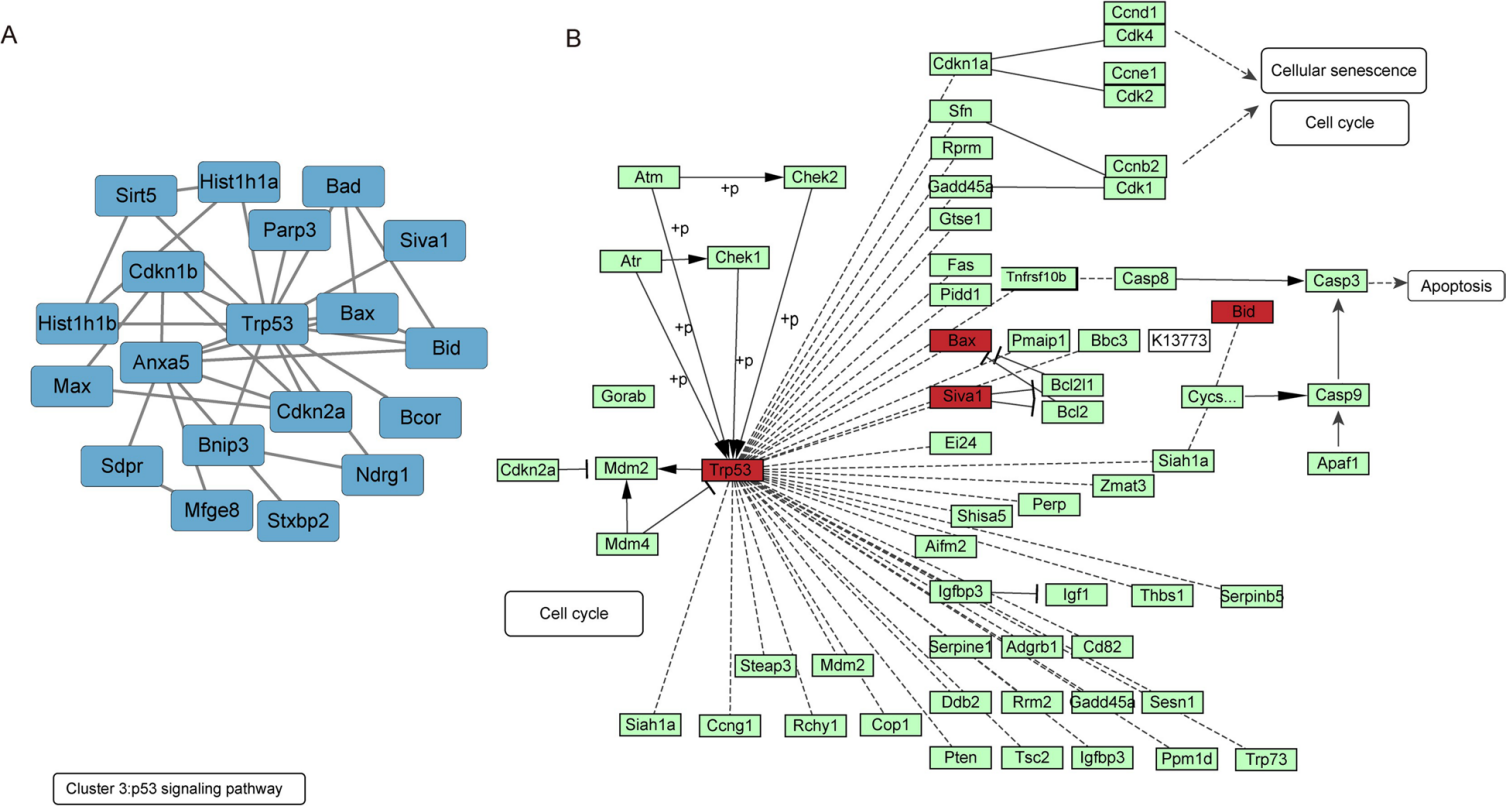
KEGG pathway enrichment analysis of cluster III proteins. (**A**) The differentially regulated cluster III protein network. The blue nodes indicate the significantly downregulated cluster III proteins (*p* < 0.05). (**B**) The position of cluster III proteins in the KEGG “p53 signaling pathway” during the NBP treatment are shown in red color

In Fig. 7A, there were 20 Proteins in clusters III including Apoptosis regulatory protein Siva (Siva1), Protein mono-ADP-ribosyltransferase PARP3 (Parp3), Histone H1.5 (Hist1h1b), NAD-dependent protein deacylase sirtuin-5 (Sirt5), Histone H1.1 (Hist1h1a), Protein max (Max), Protein NDRG1 (Ndrg1), BCL2/adenovirus E1B 19 kDa protein-interacting protein 3 (Bnip3), BCL-6 corepressor (Bcor), Bcl2-associated agonist of cell death (Bad), Syntaxin-binding protein 2 (Stxbp2), Cellular tumor antigen p53 (Trp53), Lactadherin (Mfge8), Apoptosis regulator BAX (Bax), Tumor suppressor ARF (Cdkn2a), Caveolae-associated protein 2 (Cavin2/Sdpr), BH3-interacting domain death agonist (Bid), Cyclin-dependent kinase inhibitor 1B (Cdkn1b), Annexin A5 (Anxa5). The KEGG enrichment analysis demonstrated that the top 5 enriched pathway were Apoptosis, Measles, Platinum drug resistance, p53 signaling pathway and Chronic myeloid leukemia. We pointed out the DEPs in p53 signaling pathway in Fig. 7B.

### The verification by Western blot

**Fig. 8:**
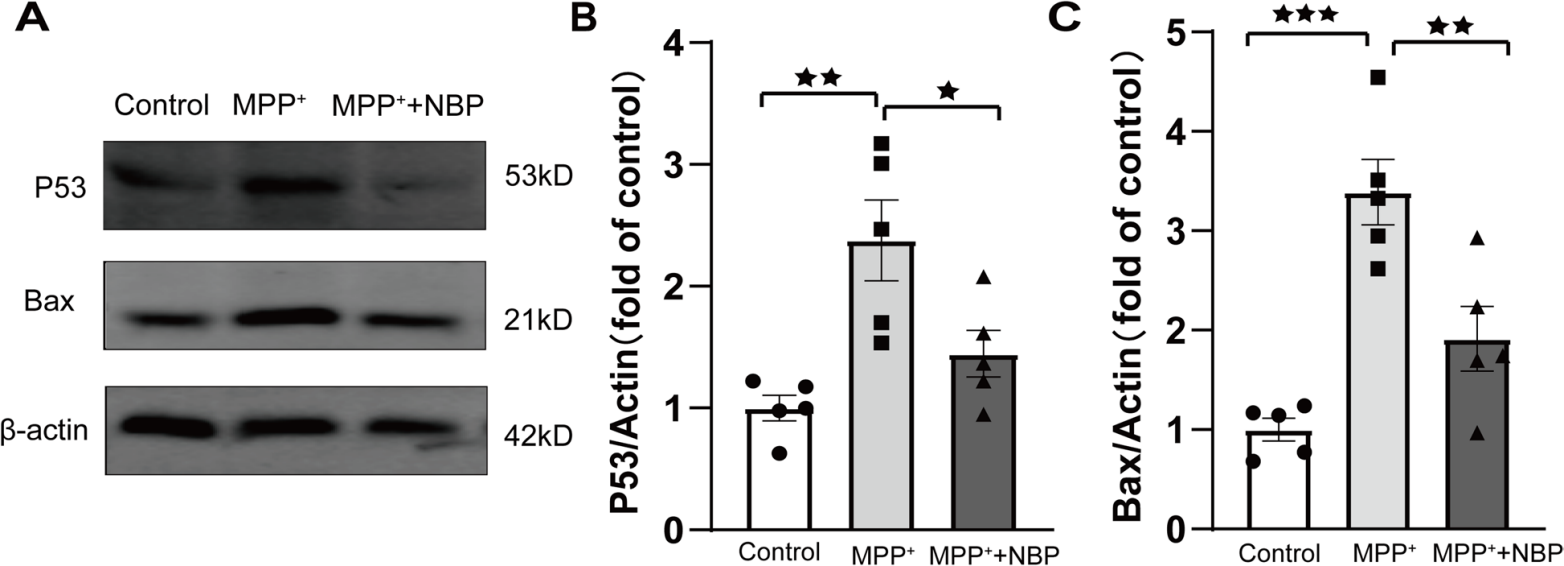
Western blot analysis of P53 and Bax. ★: *p* < 0.05; ★★: *p* < 0.01; ★★★: *p* < 0.001

Based on the result of cluster analysis, P53 and Bax were associated with “P53 signaling pathway”. Therefore, P53 and Bax were selected for Western blotting validation (Fig. 8). The result revealed that compared with control group (*n* = 5), P53 and Bax were significantly upregulated in mpp + treated group (P53:1.00 ± 0.10 vs 2.38 ± 0.33, *P* = 0.003; Bax:1.00 ± 0.11 vs 3.39 ± 0.33, *P* < 0.001), whereas the pretreatment of NBP reversed this effect (P53:1.45 ± 0.19 vs 2.38 ± 0.33, *P* = 0.035; Bax:1.91 ± 0.33 vs 3.39 ± 0.33, *P* = 0.007). The WB validation of P53 and Bax is consistent with proteomic result.

## Discussion

Parkinson’s disease (PD) is a motor related neurodegenerative disorder with cardinal clinical characters involving bradykinesias, tremor, postural instability and rigidityb [6]. The hallmarks of PD pathology are the loss of neurons in substantia nigra and the formation of Lewy bodies (LBs) in surviving neuron [5]. The aggregation of the misfolded and fibrillary α-synuclein (α-syn) are identified as the core mechanism of the formation of LBs [3]. As nigrostriatal dopaminergic pathway is primarily impaired in PD pathological process, dopamine replacement therapy based on the oral administration of L-DOPA is the first-line pharmacotherapy for PD to date [28]. In initial treatment, L-DOPA reverses these motor disturbances efficiently, whereas the long-term application may induce heterogenetic complications, particularly dyskinesia and motor fluctuations [28–30]. Therefore, the development of novel therapeutic strategies, especially those that target non-dopaminergic pathways are the urgent clinical quest.

Dl-3-n-butylphthalide (NBP; C12H14O2), extracted from the seeds of Apium graveolens, was first administrated as the therapy for patients suffered with acute ischemic stroke in 2002 in China [9, 11]. Albeit researches have documented that NBP displayed extensive pharmacological activities and exerted potentially beneficial effects in PD models both in vivo and in vitro, its precise mechanisms are still uncovered [17, 19, 31]. Therefore, we employed TMT-based LC–MS/MS to draw the differentially expressed proteins profiling of NBP pretreatment in mpp + -induced N2A cells. A total of 5828 proteins were quantified in the three replicates. Using fold-change > 1.5 and *P* < 0.05 as the cutoff to identified DEPs, compared to mpp + group, 417 proteins were determined as DEPs, among which 140 were upregulated and 277 were downregulated in mpp + -induced N2A cells with NBP pretreatment.

Subsequently, GO and KEGG analysis were performed to explore the cellular function and biological pathways enrichment of the DEPs. The result of GO-CC revealed that most DEPs were localized in cytosol and mitochondrion. As NBP held mito-protective effects on cerebral ischemia/reperfusion and cardiac ischemia models by reducing oxidative injury, alleviating mitochondrial apoptosis, and regulating mitochondrial biogenesis, we hypothesis that anti- mitochondrial injury might be one of the therapeutic approaches of NBP in PD as well. However, the exact mechanisms are required more work to illuminated. Based on the KEGG pathway enrichment analysis, “Lysosome”, “Phagosome”, “Apoptosis”, “Nucleotide metabolism”, “Ferroptosis” were all participated in the potential protective action of NBP in PD. In addition, “positive regulation of tumor necrosis factor production” and “apoptotic process” were also involved in GO-BP enrichment analysis. In this regard, the suppression of apoptotic process was supposed to be one of the salient pathways that related to the neuroprotective effect of NBP.

Next, the MCC method identified 10 hub proteins with the highest degree of connectivity, including Itgb2, Coro1a, Fermt3, Ptprc, Hcls1, Inpp5d, Ptpn6, Nckap11, Rac2 and Ncf4. Some of the proteins had been reported to be implicated in PD progress, even had been reported to be differentially expressed in PD patients. For instant, PTPRC (also named CD45) specifically dephopshorylated tyrosine residues. In AD, the deficiency of PTPRC promoted microglial activation and increased oligomeric Aβ accumulation [32, 33]. Recent study further revealed that PTPRC downregulated significantly in patients with PD [34]. ITGB2, which encoded the β2 integrin subunit, is implicated in defective adhesion and the subsequent inflammatory reaction [35]. Bioinformatic analysis revealed that ITGB2 was robust in microglia and played a role in the pathological mechanism of AD [36]. In addition, several network analyses reported ITGB2 altered in PD as well [37, 38]. Inpp5d is widely reported to be an AD risk gene [39]. Recent data processing of the available datasets further elucidated that Inpp5d was one of the hub genes involved in the overlapping molecular pathogenesis of AD and PD [37]. As Inpp5d was encoded for SHIP1, it acted as a negative regulator for PI3K/AKT signaling. Furthermore, previous study demonstrated that the depletion of SHIP1 could enhance the resistance to apoptosis [40]. Therefore, these findings indicated novel molecular mechanism of the neuroprotective effect of NBP in PD.

Cluster analysis suggested that p53 signaling pathway was one of the enriched pathways of the DEPs. In addition, p53 was also significantly differential expressed after NBP pretreatment. P53 was recognized as a tumor suppressor which encoded by the TP53 gene placed at 17p13.1 locus [41]. It was well known that p53 was essential for inducing apoptosis and was responsible for diverse cellular stresses. Pathogenic p53 integrated the cellular stresses comprising the generation ROS, inflammation, abnormal protein accumulation and Ca^2+^ overloading, to trigger cell death [42–44]. Recent study demonstrated that the specific deletion of p53 gene could eliminate dopaminergic neuronal cell death and further decrease motor deficits in MPTP-treated mice [45]. On one hand, the activated p53 interacted with antiapoptotic Bcl-2 family proteins and released Bax and Bad to open mPTP, which induced the intrinsic apoptotic pathway [46–48]. On the other hand, the accumulation of activated p53 might lead to significant impairment of autophagic clearance and promote α-syn aggregation [49]. In addition, p53 was also contributed to the regulation of DJ-1 as well as parkin both in mRNA and protein levels, which brought a feasible link between genetic and sporadic Parkinsonism in some extent [45]. Mitochondrial dysfunction was another hallmark of PD pathology. It had reported that p53 directly interacted with Parkin to inhibit its translocation to the damaged mitochondria, which aggravated the impairment of mitophagy and the consequent PD manifestations [50, 51]. In accordance with our result, NBP could break the 3-D structure of NQO1 and restrict p53 degradation in ischemia neurons [52]. Therefore, based on our result, the expression of P53 and its downstream factors including Bad and Bax were all reduced significantly after NBP pretreatment, indicating the suppression of p53 signaling pathway may be a potential therapeutic pathway for NBP. However, there is a dearth of studies to detect the association of NBP treatment and p53-medicated apoptosis in PD. Our research might shed light of novel therapeutic target of NBP in clinical PD treatment.

## Conclusion

Taken together, using TMT-based quantitative proteomics, we revealed a signature of DEPs in N2A cells with NBP pretreatment when compared to untreated N2A cells. Among DEPs, essential proteins such as Itgb2, Coro1a, Fermt3, Ptprc, Hcls1, Inpp5d, Ptpn6, Nckap11, Rac2 and Ncf4 were identified as hub genes. The KEGG pathway and PPI network analysis provided preliminary information for discovering novel therapeutic targets and the implicated pathways for NBP treatment in PD. Therefore, our study warrants further studies to comprehensively and conclusively elucidate the role of these proteins or pathways in the potential clinical applications.

## Supplementary Information

**Additional file 1.** 

**Additional file 2.**

**Additional file 3.**

**Additional file 4.**

**Additional file 5.** 

## Data Availability

The raw data has been submitted to the ProteomeX-change Consortium (http://proteomecentral.proteomexchange.org) via the iProX partner repository with the dataset identifier PXD037468. The datasets supporting the conclusions of this article are included within the article and its additional flies.
