# Supplementary material for: Proteomic Analysis of Protective Effects of Dl-3-n-Butylphthalide against mpp + -Induced Toxicity via downregulating P53 pathway in N2A Cells
Source: Proteome Sci. 2023 Jan 3;21:1. doi: 10.1186/s12953-022-00199-x (PMC9809048; doi:10.1186/s12953-022-00199-x)
Supplement: Supplementary file 1 — Additional file 1. [file 12953_2022_199_MOESM1_ESM.docx]

|  | Quantified | Identified |  |
| --- | --- | --- | --- |
| Replicate 1 | 7273 | 6970 | 95.83% |
| Replicate 2 | 6955 | 6657 | 95.72% |
| Replicate 3 | 7483 | 7167 | 95.79% |

Supplementary Table 1. The number of the quantified and identified proteins in each replicates
