# Supplementary figures and images for: Proteomic Analysis of Protective Effects of Dl-3-n-Butylphthalide against mpp + -Induced Toxicity via downregulating P53 pathway in N2A Cells
Source: Proteome Sci. 2023 Jan 3;21:1. doi: 10.1186/s12953-022-00199-x (PMC9809048; doi:10.1186/s12953-022-00199-x)

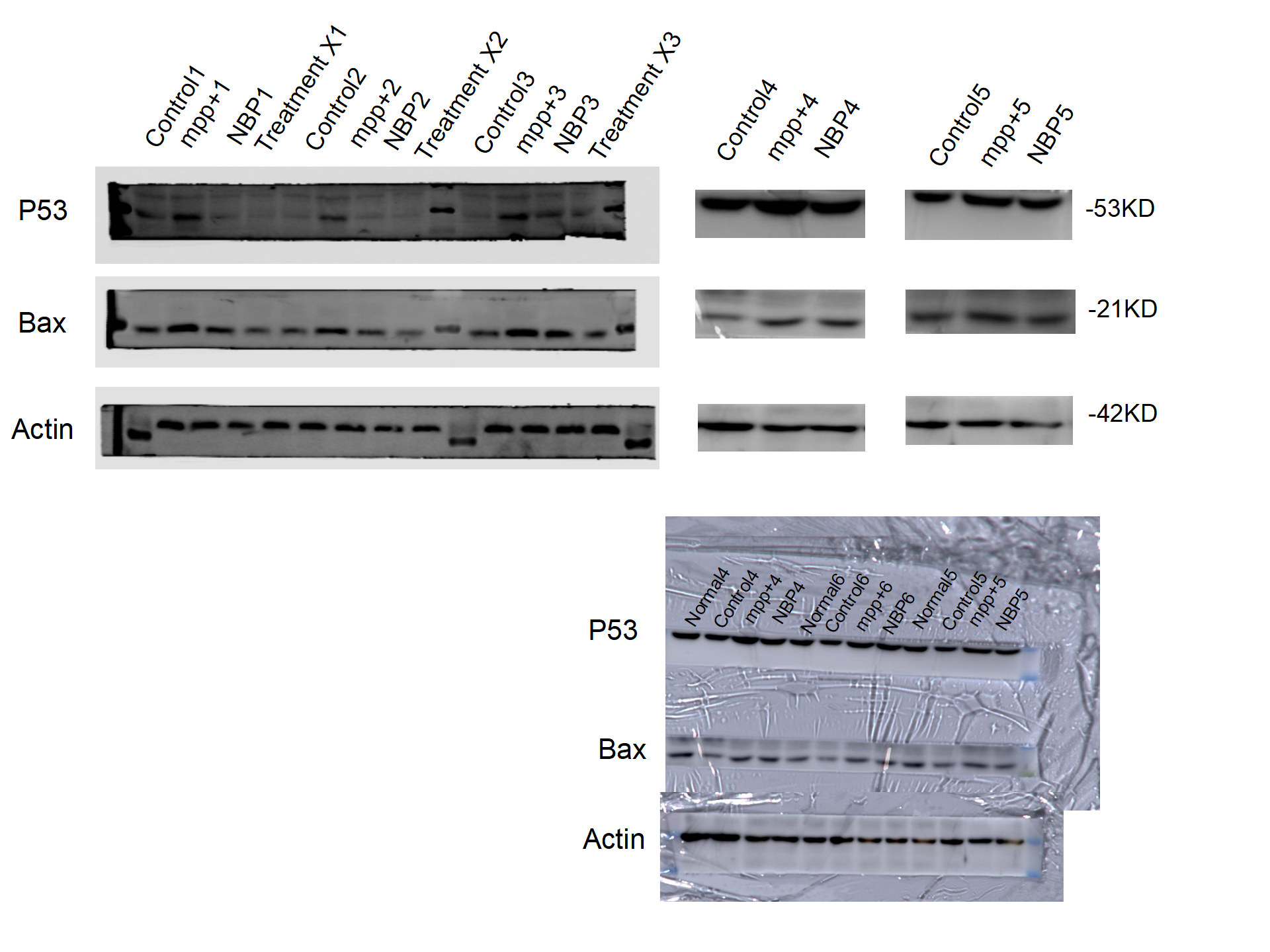

Supplement: Supplementary file 2 — Additional file 2. [file 12953_2022_199_MOESM2_ESM.tif]
